# Enhancing the value of women’s reproductive rights through community based interventions in upper Egypt governorates: a randomized interventional study

**DOI:** 10.1186/s12939-019-1042-y

**Published:** 2019-09-18

**Authors:** Ammal M. Metwally, Rehan M. Saleh, Lobna A. El-Etreby, Somia I. Salama, Ahmed Aboulghate, Hala A. Amer, Asmaa M. Fathy, Reham Yousry, Sherif E. El-Deeb, Ghada A. Abdel-Latif, Dalia M. Elmosalami, Nihad A. Ibrahim, Osama M. Azmy, Tamer Taha, Hanaa M. Imam, Mohamed Abdel Rahman, Samia A. R. Hemeda

**Affiliations:** 1 Community Medicine Research Department, National Research Centre, Giza, 12411 Egypt; 2 Community Medicine Department, Faculty of Medicine, Cairo University of Egypt, Giza, Egypt; 3 Reproductive Health Department, National Research Centre, Giza, Egypt

**Keywords:** women’s reproductive rights, Antenatal care, Natal care, Postnatal care

## Abstract

**Background:**

In 2012, the WHO described the quality of health care as the route to equity and dignity for women and children.

**Aim of the work:**

To provide community based support and empowerment to women in childbearing period to seek optimal prenatal, natal and postnatal healthcare. Achieving this is anticipated to decrease maternal morbidity and mortality in Egypt.

**Subjects and methods:**

An interventional study was conducted among women in childbearing period in the poorest two governorates of Upper Egypt. The study passed through three stages over three and a half years; pre-interventional assessment of awareness (*n* = 1000), educational interventions targeting the health providers and all women in childbearing period in their communities (*n* = 20,494), and post-intervention evaluation of change in awareness of their rights for prenatal, natal and postnatal care (no = 1150).

**Results:**

The studied indicators relating to receiving care in pregnancy, labor, and puerperium have changed dramatically as a result of the study interventions. Results of the study showed that before interventions, the surveyed women had inaccurate knowledge regarding most of the items related to their rights. The percentages of women aware of their right to have pregnancy card increased and those who possessed a pregnancy card were doubled with a significant percent change of more than 25%. Some indicators showed more than 75% improvement, including; percent of surveyed women who knew that it’s their right to follow up their pregnancy and to deliver with a specialized doctor, a trained nurse or at an equipped health facility, and those who knew their right to have at least two home preparations necessary for safe delivery at home.

**Conclusion and recommendations:**

More work is needed in order to achieve the targeted reduction of maternal mortality. This could be achieved by ensuring accessible and high quality care provided by the governmental health facilities together with increasing the awareness of women regarding their rights in receiving such care.

## Background

Reproductive rights have been recognized as one of the fundamental human rights [1]. According to the World Health Organization (WHO), reproductive rights rest on the recognition of the right of all couples and individuals to decide freely and responsibly the number, spacing and timing of their children and to have the information and means to do so, and the right to attain the highest standard of sexual and reproductive health. They also include the right of all to make decisions concerning reproduction, free of discrimination, coercion and violence [2]. Reproductive rights began to develop as a subset of human rights at the United Nation’s 1968 International Conference on Human Rights [3]. Reproductive rights were clarified and endorsed internationally in the Cairo Consensus that emerged from the 1994 International Conference on Population and Development [4]. Adequate reproductive health and women’s reproductive right help to reduce poverty, promote economic growth, raise female productivity, lower fertility, and improve child survival and maternal health. Utilizing the reproductive right can decrease pregnancy related morbidity and mortality and give women the autonomy to make decisions about their lives including their reproductive life [5]. This could be a step to achieve the millennium development goal number five which focuses on improving maternal health including eliminating inequity by ensuring universal access to maternal health services [6]. In Egypt, the status of reproductive health and the quality of life of females are not satisfactory. There is still a lot of challenges to be met; unequal access to information, care and basic health services, early marriage, deeply-rooted believes, and illiteracy leading to lack of awareness and underutilization of reproductive rights among women [7]. In addition, another barrier for utilizing antenatal care (ANC) natal care and postnatal care was found to be the poor quality of health care provided by their health providers which undermines the trust people have in those services [8]**.** In another study, Egyptian women who did not receive ANC were found to have up to 10 times higher risk of deaths compared to those who received ANC [9]**.**

The current study provides a series of interventions aiming to enhance women’s rights and eventually decrease maternal morbidity and mortality in Egypt. The study uses a deductive approach to test the hypothesis that community based interventions targeting women in childbearing period to enhance reproductive rights could raise the awareness and increase the utilization of the governmental health services.

While the data for the current study was gathered in Egypt, its findings could be a useful resource and have implications for government and primary health care providers across many countries given similarities in their cultures and government responses.

## Methods

### Study setting

This study targeted pregnant and postpartum women in 21 villages and 119 satellites of two governorates of Upper Egypt; Al Fayoum and Benisuef; 11 villages and 76 satellites belonging to Al Fayoum governorate (in Senores and Youssef El Sedeek districts) and 10 villages and 43 satellites belonging to Benisuef governorate (in El Fashen and Somosta districts). These two governorates of Upper Egypt were ranked the poorest as declared in Egypt report of 2015 [6]**.**

The selection of districts and villages for the study conduction was done through participatory approach with local governmental authorities. The selection was aiming to decrease the documented disparity between rural and urban communities. There is a significant disparity between urban and rural communities for both maternal mortality ratio (MMR) as well as the awareness and perception about maternal mortality in Egypt [6, 7, 9]**.** In 2013, the national MMR in Egypt was found to be 43.5 deaths per 100 thousand births with the highest rate for the rural communities of upper and lower Egypt governorates ranging between 60 and 65 deaths per 100,000 live births; while lowest rates were observed in city governorates ranging between 24 and 37 deaths per thousand births [6, 7]**.** Furthermore, awareness and care seeking behaviors about maternal mortality issues were found to be poor in rural communities compared to urban ones [9]**.**

### Study design and participants’ characteristics

The study was a randomized intervention evaluation study for which the interventions were conducted along three and a half years starting from August 2012 till March 2016. The study passed through three stages; pre-interventional assessment of women awareness of their rights, community based interventions targeting primary health care providers, community health workers and women in their communities, and post-intervention evaluation of the change in the women’s awareness of their reproductive rights.

The second target group was the host community which refers to the individuals living in the area of the study, their leaders, and community-based organizations that serve or represent them directly who are the paramedical and medical health providers.

### Study phases

This study was done over three phases:

The first phase included: assessing the level of beneficiaries’ awareness, attitude and behavior towards their rights in standard of care and treatment which was conducted along six months starting from February 2012 till August 2012.

The second phase of the study included community based interventions where three types of interventions were conducted along three years starting August 2012 till March 2016.

The third phase included: evaluation of the impact of the interventions on change of the level of beneficiaries’ awareness, attitude and behavior towards their rights in standard of care and treatment and on the utilization of the governmental health services. The evaluation was conducted along six months starting from April 2016 till October 2016.

Both the assessment and the evaluation phases were done by the community health workers after their training on how to conduct the survey under supervision of a professional team of community medicine and reproductive health departments of the National Research Centre.

The target beneficiaries were women in childbearing period with special focus on current pregnant women, women in postpartum period and newly married women. The women targeted during the assessment phase were representative sample (*n* = 1000 women), whereas during intervention 85% of women in childbearing period were targeted (*n* = 20,494). Then another subsample of women (*n* = 1150) were randomly targeted to measure the impact of the interventions on the change of awareness and utilization of the services during the evaluation phase.

The targeted women during the assessment were different from those who were targeted during the evaluation in order to avoid any information bias for the asked questions.

### Sample size and sampling technique

The sampling technique was used during both the pre- and post-intervention. The sample included women in their reproductive age who are either currently pregnant women or women in post-partum period or newly married women as these women will be the direct beneficiaries of the governmental reproductive health services. These groups were selected as they were expected to benefit most from the study interventions.

Both the pre- and post-intervention phases were done to measure the level of awareness of as well as the utilization of the health facilities before and after the interventions to measure their impact.

- Two-stage random sampling was used; households were randomly selected, and then one woman of reproductive age was randomly selected from each household. Sample size was calculated to meet the Division of Reproductive Health (DRH) standards at the center for disease control and prevention of point estimates within +/− 5% of the true population prevalence, with 95% confidence and estimated response rate of 80% [10]**.** Accordingly, in order to have completed interviews for at least 480 women of reproductive age, at least 550 households were contacted from each governorate. This estimate was based on household lists where only households with women of reproductive age were identified. Household lists, having current pregnant women, newly married women and women in the postpartum period were obtained from community health workers (CHWs) zones within the targeted villages that were used in sampling.
- The samples were chosen from the targeted villages with a range of 2–8 interviewers per village according to beneficiaries’ size within each village. For each interviewer a range of 14 to 17 households were surveyed. The households were randomly selected. The first household was selected from the random table by the supervisor. The second household was selected by adding the sampling interval to the original random number and so on till interviewer target was met.
- During the pre-intervention phase, out of 550 targeted women for survey from each governorate, 480 and 520 women in El Fayoum and Benisuef governorates respectively completed the survey and were included in the study with response rate of 87 and 94.5%. During the post-intervention phase, out of 550 targeted women for survey from each governorate, 480 and 520 women in El Fayoum and Benisuef governorates respectively completed the survey and were included in the study with response rate of 87 and 94.5%.

### Description of the community based intervention

1. Educational and promotional materials were developed keeping in view the users and the respondents (beneficiaries) in the community. All the developed and produced materials were distributed to cover all the targeted health units within the targeted districts. The developed materials included; two educational pockets modules; one targeting nurses and the other targeting CHWs. The module targeting CHWs included mainly drawings, illustrations and role plays. Four types of promotional materials were developed to disseminate information to the targeted women. The developed promotional materials included the following: calendar, three wall posters, “Who will win with us” game (derived from “Who will win a million” / “Who wants to be a millionaire”) and drama production on CDs. The theme of the developed messages for the drama’s scenario is what women and their families need to know regarding their rights in receiving care during antenatal, natal, and postnatal period. It also included all the services that are provided within the primary health facilities according to the period of continuum of care and how to recognize danger signs, where to go for delivery care and emergency care and how to arrange in advance for transport.
2. A 103 nurses and 63 CHWs were trained after being assessed for their knowledge, attitude and behaviors related to maternal and obstetric care. They were chosen because this group (CHWs) is proved to play an important role in the life of women in the rural and urban low-income communities in Egypt [11, 12]**.** Their training was followed by conduction of refresher courses for the promotion of good antenatal, natal and post-natal care for the nurses and CHWs. During the training courses much emphasis was put on identifying women’s rights for having quality care, recognition of danger signs during pregnancy, labor and puerperium. During the refresher courses the developed promotion materials were used as aiding materials to deepen and sustain the gained knowledge.
3. Outreach and in facility education sessions by both the trained nurses and CHWs were conducted with at least one month home visit for every woman. Four campaigns were conducted yearly with 3 months interval, with a total of 14 campaigns. The promotional materials were regularly distributed during campaigns and home visits along the three and a half years of intervention.

### Data collection types and tools during the first and the third phase

The data-collection instrument used was a structured questionnaire that was derived from that of the Center for Disease Control (CDC), 2010 [13]**,** as well as from the information given to the pregnant women having the pregnancy card during their ANC visits which is designed and authorized by the Maternity and Childhood sector in the Ministry of Health in Egypt. A structured interview was done to collect the necessary data by the CHWs under the supervision of the team. The questionnaire focused on eight indicators to detect women’s awareness about ANC and pregnancy complications, six indicators to detect women’s awareness about delivery care and complication and three indicators to assess women’s awareness about postpartum care and complications.

### Statistical analysis

All completed questionnaires were statistically analyzed with SPSS, version 16. Data were presented by percentages. The change in the knowledge and practices were the basic instruments for comparison. Z test between proportions was used for comparison. The analysis was done using Z test between two proportions [14]. *P* value < 0.05 was considered significant. The impact of the intervention was calculated by the percent change between the assessment and evaluation indicators. The percent change for each indicator was calculated by deduction of the indicator percentage value during the assessment from that during the evaluation.

### Indicators used during the assessment and evaluation

Five health care access indicators (4 awareness and 1 practice) including the percentage of surveyed women who knew:

1. their right to issue a health card once they know they are pregnant
2. that the health card is obtained from the health unit.
3. their right to obtain a health card for free
4. who actually possess a health card (practice)
5. their right to obtain at least two services out of the contents of the health card

Six prenatal care indicators including: The percentage of women who knew:

1. their right to follow up their pregnancy with a specialized doctor, a trained nurse or at the health unit
2. their right to follow up their pregnancy not less than four times at the health unit
3. at least two of their rights in health care during follow up at the health unit
4. at least two of their rights for having the laboratory investigations they should do at the heath unit at the beginning of pregnancy
5. their right to attend health education sessions at the health unit
6. at least two pregnancy complications which give them the right to go to the hospital immediately

Seven postpartum indicators including: the percentage of women who knew:

1. their right to deliver in a hospital (or any equipped medical center)
2. their right to have their delivery attended by a specialized doctor or a trained nurse
3. their right to have their delivery attended by a specialized doctor and in a hospital when experienced prolonged delivery
4. their right to have at least two home preparations necessary for safe delivery when delivery is attended at home
5. at least two delivery complications which give them the right to go to the hospital immediately
6. their right to be visited during the puerperium by the nurse after reporting the delivery
7. at least two complications during the puerperium which give them the right to go to the hospital immediately

### Ethical considerations

The study complied with the International Ethical Guidelines for Biomedical Research Involving Human Subjects [15]. The Research and Ethical Committee of the National Research Centre have cleared the study protocol with the ethical approval registration number 10140. Informed consent was obtained from all participants involved in the study and the information obtained at the individual level was kept strictly confidential.

## Results

Awareness of the right of women to receive care at the health units is presented in Table 1. It was generally unsatisfactory but was substantially improved after health education. The percent change in awareness was more or less the same for both governorates. The percent change in awareness was more than 40% for four out of five indicators; awareness of their right to issue a health card once they know they are pregnant, knowing that the health card is obtained from the health unit, awareness of their right to obtain a health card for free, awareness of their right to obtain at least two of the contents of the health card. The least percent change in awareness was noticed in the percentage of surveyed women who actually possess a health card (26.9%). Awareness of the right of women to issue a health card once they know they are pregnant was the least known item by the interviewed women in the two governorates especially in El Fayoum (7.4% in El Fayoum versus 16.4% in Benisuef), while it showed the highest percentage change after the intervention (81.1%).

**Table 1:** Evaluation of women’s right indicators for receiving care at the health units in the targeted villages of the selected governorates

Indicator	El Fayoum Governorate			Benisuef Governorate			Total		
	Before *n* = 520	After *n* = 543	% Change	Before *n* = 480	After *n* = 607	% Change	Before n = 1000	After n = 1150	% Change
	N (%)	N (%)	%	N (%)	N (%)	%	N (%)	N (%)	%
First: Health Card									
1. % of surveyed women who know their right to issue a health card once they know they are pregnant	37 7.4%	505 93.0%	85.6%^*^	82 16.4%	565 93.1%	76.7%^*^	119 11.9%	1070 93.0%	81.1%^*^
2.% of surveyed women who know that the health card is obtained from the health unit	171 35.6%	440 81.0%	45.4%^*^	127 24.4%	498 82.0%	57.6%^*^	298 29.8%	938 81.6%	51.8%^*^
3.% of surveyed women who know their right to have their delivery attended by a specialized doctor and in a hospital when experienced prolonged delivery	126 26.3%	410 75.5%	49.2%^*^	139 26.7%	483 79.6%	52.9%^*^	265 26.5%	893 77.7%	51.2%^*^
4.% of surveyed women who actually possess a health card (from a total of 827 women exposed to pregnancy;363 before and 464 after)	143 39.4%	308 66.1%	26.7%^*^	229 49.4%	442 76.6%	27.2%^*^	372 45.0%	750 71.9%	26.9%^*^
5.% of surveyed women who know their right to obtain at least two services out of the contents of the health card^a^	64 13.3%	295 54.3%	41.0%^*^	72 13.8%	354 58.3%	44.5%^*^	136 13.6%	649 56.4%	42.8%^*^

*z test between two proportion, *P* < 0.05 is significant

^a^The most frequent two were: Health Card Contents: 1-Personal data (name, age, address) 2- Health status (e.g. weight, height, blood pressure, blood sugar) 3- History and circumstances of previous deliveries 4- Visits made to health unit and their results

Participants were found to have limited knowledge regarding most of the items relating to their right to receive pregnancy care and follow up in the health units as shown in Table 2. Awareness of the right of women to follow up their pregnancy with a specialized doctor, a trained nurse or at the health unit was the most known item in the two governorates especially in El Fayoum (86.5% in El Fayoum versus 66.5% in Benisuef). On the other hand, awareness of the right of women to attend health education sessions at the health unit was the least known item by the interviewed women in both governorates especially in El Fayoum (20% in El Fayoum versus 31.2% in Benisuef). The surveyed women in Benisuef had better knowledge compared to those in El Fayoum regarding knowing at least two of their rights in health care during follow up at the health unit and their right to attend health education sessions at the health unit. On the contrary, women in El Fayoum had better knowledge compared to those in Benisuef regarding their right to follow up their pregnancy with a specialized doctor, a trained nurse or at the health unit. General improvement in awareness of the surveyed women was noticed especially the awareness of their right to attend health education sessions at the health unit and knowing at least two pregnancy complications which give them the right to go to the hospital immediately.

**Table 2:** Evaluation of women’s right indicators for receiving pregnancy care and follow-up at the health units in the targeted governorates

Indicator	El Fayoum Governorate			Benisuef Governorate			Total		
	Before *n* = 520	After *n* = 543	Rate of Change	Before *n* = 480	After *n* = 607	Rate of Change	Before *n* = 1000	After *n* = 1150	Rate of Change
	N (%)	N (%)	%	N (%)	N (%)	%	N (%)	N (%)	%
Second: Pregnancy Care and Follow- Up									
1. % of surveyed women who know their right to follow up their pregnancy with a specialized doctor, a trained nurse or at the health unit^a^	256 86.5%	421 90.3%	3.8%	231 66.5%	500 86.25%	19.8%^*^	487 75.7%	921 88.0%	12.3%
2. % of surveyed women who know their right to follow up their pregnancy not less than four times at the health unit**	210 43.8%	413 76.1%	32.3%^*^	231 44.4%	346 57.0%	12.6%^*^	441 44.1%	759 66.0%	21.9% ^*^
3. % of surveyed women who know at least two of their rights in health care during follow up at the health unit ^a^	233 48.5%	383 70.5%	22.0%^*^	331 63.7%	436 71.8%	8.1%	564 56.4%	819 71.2%	14.8%^*^
4.% of surveyed women who know at least two of their rights for having the laboratory investigations they should do at the heath unit at the beginning of pregnancy^b^	174 36.2%	366 67.4%	31.2%*	185 35.6%	336 55.4%	19.8%^*^	359 35.9%	702 61.0%	25.1%^*^
5.. % of surveyed women who know their right to attend health education sessions at the health unit	96 20.0%	260 47.9%	27.9%*	162 31.2%	411 67.7%	36.5%*	258 25.8%	671 58.3%	32.5%*
6. % of surveyed women who know at least two pregnancy complications which give them the right to go to the hospital immediately^c^	185 38.5%	406 74.8%	36.3%*	195 37.5%	395 65.1%	27.6%*	380 38.0%	801 69.7%	31.7%

*z test between two proportion, *P* < 0.05 is significant

^a^ The most frequent mentioned two: Women’s rights in health care during follow-up:

1-weight and height measurement 2-laboratory investigations 3- tetanus vaccination 4- sugar analysis 5- X-ray

^b^The most frequent mentioned two: Women’s rights in the laboratory investigations they should do at the beginning of pregnancy: complete urine analysis – blood analysis – determination of blood group and Rh factor – thyroid gland tests

^c^The most frequent mentioned two: Pregnancy complications which give woman the right to go to the hospital immediately: severe headache and blurring of vision – rise of body temperature – convulsions – foul smelling vaginal discharge – vaginal bleeding even in small amounts or without pain

Table 3 presents knowledge about different issues relating to the right of women to receive care during delivery and puerperium at the health units. It was found to be generally unsatisfactory but was improved after health education. Awareness of the right of women to have their delivery attended by a specialized doctor or a trained nurse and to have their delivery attended by a specialized doctor and in a hospital in case of prolonged delivery were the most known items by the interviewed women in the two governorates. On the other hand, awareness of the right of women to have at least two home preparations necessary for safe delivery in case of delivery at home was the least known item by the interviewed women in both governorates especially in El Fayoum (7.1% in El Fayoum versus 18.3% in Benisuef). General improvement in awareness of the surveyed women was noticed especially the awareness of the right of women to have at least two home preparations necessary for safe delivery in case of delivery at home. The surveyed women in El Fayoum showed more improvement in awareness of all items compared to women in Benisuef except their knowledge of at least two complications during the puerperium which give them the right to go to the hospital immediately.

**Table 3:** Evaluation of women’s right indicators for receiving care during delivery and puerperium at the health units in the targeted governorates

Indicator	El Fayoum Governorate			Benisuef Governorate			Total		
	Before *n* = 520	After *n* = 543	% Change	Before *n* = 480	After *n* = 607	% Change	Before *n* = 1000	After *n* = 1150	% Change
	N (%)	N (%)	%	N (%)	N (%)	%	N (%)	N (%)	%
Third: Care During Delivery and Puerperium									
1.% of surveyed women who know that it’s their right to deliver in a hospital (or any equipped medical center)	221 46.0%	439 80.8%	34.8%*	192 36.9%	364 60.0%	23.1%*	413 41.3%	803 69.8%	28.5%*
2.% of surveyed women who know their right to have their delivery attended by a specialized doctor or a trained nurse	326 67.9%	488 93.5%	25.6%*	340 65.4%	442 82.9%	17.5%*	666 66.6%	930 88.2%	21.6%*
3.% of surveyed women who know that in case of prolonged delivery it’s their right to have their delivery attended by a specialized doctor and in a hospital	296 61.7%	509 93.7%	31.8%.*	360 69.2%	523 86.2%	17.0%*	656 65.6%	1032 89.7%	24.1%*
4.% of surveyed women who know their right to have at least two home preparations necessary for safe delivery when delivery is attended at home ^a^	34 7.1%	512 94.3%	87.2%*	95 18.3%	530 87.3%	69.0%*	129 12.9%	1042 90.6%	77.7%*
5.% of surveyed women who know at least two delivery complications which give them the right to go to the hospital immediately^b^	185 (38.5)	406 74.8%	36.3%*	195 37.5%	395 65.1%	27.6%*	380 38.0%	801 69.7%	31.7%*
6.% of surveyed women who know their right to be visited during the puerperium by the nurse after reporting the delivery	210 43.8%	413 76.1%	32.3%*	231 44.4%	346 57.0%	12.6% *	441 44.1%	759 66.0%	21.9%*
7.% of surveyed women who know at least two complications during the puerperium which give them the right to go to the hospital immediately^c^	154 32.1%	366 67.4%	35.3%*	94 18.1%	420 69.2%	51.1%*	248 24.8%	786 68.3%	43.5%*

*z test between two proportion, *P* < 0.05 is significant

^a^The most frequent mentioned two: Home preparations for safe delivery: choose the delivery room such that it is clean and well-ventilated– proving clean clothes and clean bed covers for the mother’s bed

^b^The most frequent mentioned two: Danger signs during delivery which give the woman the right to go to the hospital immediately: breach or hand or cord presentation – bleeding before the descent of the baby’s head – uterine contractions for more than ten hours – fainting – convulsions

^c^The most frequent mentioned two: Danger signs during the puerperium: rise of body temperature (puerperal fever) – increasing hemorrhage – attacks of convulsions or fainting

## Discussion

The topic of women′s reproductive rights is a debated issue in politics and social sciences. However, while many scholars focus on women′s issues relating to human rights globally, little research has been done on reproductive rights in Egypt especially the right of Egyptian women to have quality antenatal, natal, and postnatal care and to know about those rights.

Over the past 25 years, Egypt has recorded important achievements in improving maternal survival and health. Progress in coverage of prenatal care in Egypt has also been noteworthy in the last decade. In 2014, around 90% of mothers underwent antenatal care checks during pregnancy, 83% of them having had antenatal care on a regular basis. Among all births, 92% were attended by a skilled birth attendant and 87% took place in a health facility [16]**.**

Despite these significant improvements, regional disparities remain substantial in the most disadvantaged areas of the country, especially in rural areas. Substantial inequalities persist also across socio-economic groups. Maternal mortality followed a similar declining trend, from 174 maternal deaths per 100,000 live births in 1992 to 52 maternal deaths per 100,000 live births in 2013 [17]**.**

Findings of the present study revealed poor knowledge regarding important reproductive health rights which should be an area of concern for policy makers. In the current study, surveyed women had defective knowledge regarding most of the items relating to their right in receiving care in the health units especially their right to issue a health card once they knew they were pregnant (11.9%). Also, less than half of them knew their right to follow up their pregnancy not less than four times at the health unit (44.1%). This could be attributed to limited access to health services, poor educational level, low socioeconomic status and repeated pregnancies; all of which could undermine the ability of these women to be aware of and make use of their reproductive rights. The denial of these rights not only harms these women but also affects the whole community and eventually the country development. This emphasizes the need for community interventions in order to enhance women’s reproductive rights and empower women to be fully aware of them and be able to exercise these rights.

Concerning the right of women to receive pregnancy care and follow up at the health units, women in the present study showed more knowledge especially regarding their right to follow up their pregnancy with a specialized doctor, a trained nurse or at the health unit (75.7%). These findings agreed with the results of a Nepalese study [18].

Knowledge of women about their right to receive care during delivery and puerperium in our study was generally unsatisfactory as only 40% of them knew their right in institutional delivery and about half of the women knew their right in receiving postnatal care. When compared to similar studies these percentages would be considered low as in a Nepalese study where 80% of women were aware of their rights in institutional delivery and about three quarters knew their need for post-natal care visits and their right to have such visits [19]. Similar results were observed in other studies [20, 21]**.**

Other factors contributing to poor knowledge of the women about their rights during antenatal, natal, and post-natal care include early age of marriage, less exposure to the media, having conservative traditions isolating them from the outside world leading to lack of access to information and eventually lower level of awareness.

This may imply the need for legislations to define and protect women’s reproductive rights given that the Egyptian Constitution comprises articles that ensure the protection of women against violence, torture, mutilation, organ trade, violence, sexual exploitation and assault, and human trafficking with no clear entity for the protection of reproductive rights [22]. It is equally important to enforce these laws and make them well known to the public.

After the intervention, results of the study showed that the majority of indicators have improved. Some indicators showed improvement of more than 75%, other indicators showed improvement from 50 to 75% which necessitate more work in order to reach the required achievement. Only one indicator showed improvement less than 50% which is the percent of surveyed women who know that it is their right to attend health education sessions at the health unit only in El Fayoum governorate and this indeed needs to be changed.

## Conclusion and recommendations

Despite the great progress achieved in many areas relating to maternal mortality, reproductive rights of women need to be addressed more closely in terms of accessibility to all women especially the poor rural women. Also awareness of these rights and how to use them must be conveyed and taught to these women to enable them to exercise these rights. This will promote women’s health which will be reflected on her family and on the country development as a whole.

The study showed that before the project intervention the surveyed women had defective knowledge regarding most of the items relating to their rights in receiving care in pregnancy, labor and puerperium. The studied indicators have changed dramatically as a result of the applied interventions. This reflects the necessity of the continuity of educational interventions to the poor underprivileged communities.

More work is needed in order to reach the required achievement for maternal mortality reduction through ensuring accessible and high quality care provided by the governmental health facilities to increase access to skilled routine and emergency obstetric care and also implementing a prenatal surveillance program, which will help to monitor the quality and frequency of prenatal care visits together with increasing the awareness of women regarding their rights in receiving such care and subsequently decreasing the disparity for awareness as well as the mortality rates between governorates.

## Data Availability

The datasets used and/or analysed during the current study are available from the corresponding author on reasonable request.

## References

[1] International Committee on Population and Development. Plan of action 1994. http://lao.unfpa.org/www.un.org/popin/icpd/conference/offeng/poa.html. Accessed 17 Jul 2019.

[2] Gender and reproductive rights. World Health Organization. 2015. http://who.int/reproductivehealth/en/. Accessed 13 Jun 2019.

[3] Freedman LP, Stephen LI (1993). Human rights and reproductive choice. Stud Fam Plan.

[4] Reproductive rights: advancing human rights. United Nations Population Fund (UNFPA). http://www.unfpa.org/human-rights. Accessed 3 May 2019.

[5] Sharma S (2004). Reproductive rights of Nepalese women: current status and future directions. KUMJ..

[6] Trends in maternal mortality: 1990 to 2013. Estimates by WHO, UNICEF, UNFPA, The World Bank and the United Nations Population Division. World health Organization. 2014. https://apps.who.int/iris/bitstream/handle/10665/112682/9789241507226_eng.pdf;jsessionid=600D59FBF919EF4911F470E715C3A79B?sequence=2 Accessed 7 Aug 2019.

[7] Egypt’s progress towards Millennium Development Goals. World Health Organization. 2015. https://www.eg.undp.org/content/dam/egypt/docs/Publications/Docs%20MDGs/Final%20MDG%20English%202015.pdf. Accessed 7 Aug 2019.

[8] Metwally AM, Abdel-Latif GA, Saleh RM, Elmosalami DM, Ibrahim NA, Imam HM (2014). Empowerment of medical and paramedical health providers role for achieving the millennium development goal of reducing maternal mortality in Egypt. Eur J Sci Res.

[9] Metwally AM, Abdel-Latif GA, Salama SI, Tawfik A, Elmosalami DM, AbdelMohsen AM (2013). Care seeking behaviors of rural women in Egypt: community based study. J Appl Sci Res.

[10] Fleiss JL, Levin B, Paik MC (2003). Statistical methods for rates and proportions. Third edition.

[11] Metwally AM, Ibrahim NA, Saad A, Abu El-Ela MH (2006). Improving rural women role in health and environmental issues. Int J Environ Health Res.

[12] Metwally AM, Saad A, Ibrahim NA, Emam HM, El-Etreby LA (2007). Monitoring progress of the role of integration of environmental health education with water and sanitation services in changing community behaviours. Int J Environ Health Res.

[13] Assessment Toolkit for conflict-affected women. Division of Reproductive Health, National Center for Chronic Disease Prevention and Health Promotion, Coordinating Center for Health Promotion, Department of Health and Human Services, CDC. http:// www.cdc.gov/reproductivehealth/Global/PDFs/Intro_TAG508.pdf. .

[14] Z-statistics vs. T-statistics. Khan Academy. 2018. https://www.khanacademy.org/math/statistics-probability/significance-tests-one-sample/more-significance-testing-videos/v/z-statistics-vs-t-statistics. Accessed 22 Feb 2018.

[15] International Ethical Guidelines for Biomedical Research Involving Human Subjects. CIOMS/WHO. 1993. 14983848

[16] Ministry of Health and Population [Egypt], El-Zanaty and Associates, and ICF International (2015). Egypt demographic and health survey 2014.

[17] Maternal mortality bulletin. Ministry of Health and Population [Egypt], WHO, UNICEF, UNFPA, World Bank Group, and United Nations Population Division. 2014. https://www.unicef.org/egypt/sites/unicef.org.egypt/files/2018-04/MoHP_2014_%20Maternal_Mortality_Bulletin.pdf. Accessed 6 Aug 2019.

[18] Maskey KP (2008). Reproductive health status of people of Duwakot VDC Bhaktpur district. Nursing Magazine of Nepal Nursing Council.

[19] Kaphle M (2013). Awareness and utilization of reproductive rights among the women of reproductive age in Kapan VDC, Nepal. JHAS..

[20] Rawal LB, Tiwari SK, Devkota BS, Chaulagain M, Upadhaya R, Kanda K (2004). Women’s educational status and maternal and child health care practices in Jumla district, West Nepal. J Nepal Health Res Counc.

[21] Barkat A, Majid M (2003). Adolescent reproductive health in Bangladesh: status, policies, programs, and issues.

[22] Constitution of the Arab Republic of Egypt. 2014. https://www.egypt.gov.eg/arabic/laws/download/Constitution_2014.pdf .

